# Phase Formation Behavior and Thermoelectric Transport Properties of *P*-Type Yb_x_Fe_3_CoSb_12_ Prepared by Melt Spinning and Spark Plasma Sintering

**DOI:** 10.3390/ma13010087

**Published:** 2019-12-23

**Authors:** Kyu Hyoung Lee, Sang Hyun Bae, Soon-Mok Choi

**Affiliations:** 1 Department of Materials Science and Engineering, Yonsei University, Seoul 03722, Korea; khlee2018@yonsei.ac.kr; 2 School of Energy, Materials and Chemical Engineering, Korea University of Technology and Education, Cheonan 31253, Korea; khansh@koreatech.ac.kr

**Keywords:** multiple phases, thermoelectric, skutterudite, melt spinning, heterograin

## Abstract

Formation of multiple phases is considered an effective approach for enhancing the performance of thermoelectric materials since it can reduce the thermal conductivity and improve the power factor. Herein, we report the in-situ generation of a submicron-scale (~500 nm) heterograin structure in *p*-type Yb-filled (Fe,Co)_4_Sb_12_ skutterudites during the melt spinning process. Mixed grains of Yb_x_Fe_3−y_Co_1+y_Sb_12_ and Yb_z_Fe_3+y_Co_1−y_Sb_12_ were formed in melt spun ribbons due to uneven distribution of cations. By the formation of interfaces between two different grains, the power factor was enhanced due to the formation of an energy barrier for carrier transport, and simultaneously the lattice thermal conductivity was reduced due to the intensified boundary phonon scattering. A high thermoelectric figure of merit *z*T of 0.66 was obtained at 700 K.

## 1. Introduction

Skutterudite-based compounds such as *n*-type Co_4_Sb_12_-based and *p*-type (Fe,Co)_4_Sb_12_-based alloys are promising candidates for medium-high temperature (hot side temperature *T*_hot_ ~ 500 °C) thermoelectric (TE) power generation applications [1,2,3]. The maximum efficiency (*η*_max_) of a TE power generation system is expressed by the following Equation (1):

(1) $$\eta_{\max}=\left[\frac{T_{H}-T_{C}}{T_{H}}\right]\left[\frac{{\left(1+zT_{\mathrm{avg}}\right)}^{1/2}-1}{{\left(1+zT_{\mathrm{avg}}\right)}^{1/2}+\left(T_{C}/T_{H}\right)}\right],$$

where *T*_H_ and *T*_C_ are the hot side temperature and cold side temperature of TE legs (*p*- and *n*-type TE materials in module), and *zT*_avg_ is the optimum dimensionless figure of merit *zT* (= *S*^2^*σT*/*κ*, where S is the Seebeck coefficient, *σ* is the electrical conductivity, and *κ* is the total thermal conductivity at a given absolute temperature *T*) value of the TE legs at average temperature (*T*_avg_ = (*T*_H_ + *T*_C_)/2). Thus the development of skutterudites with a high *zT* is a prerequisite to realize a highly-efficient medium-high temperature TE power generation system with economic feasibility.

One of the most effective approaches to enhance the *zT* of skutterudites is the introduction of filler atoms including alkali, alkaline earth, and rare earth metals in nanocages of the lattice, which triggers a rattling effect to intensify high-frequency phonon scattering. Significantly reduced lattice thermal conductivity (*κ*_lat_ = *κ* − *κ*_ele_, where *κ*_ele_ is the electronic contribution of thermal conduction) was obtained both in *n*-type and *p*-type skutterudites [1,4]. Recently, based on the resonant scattering concept [5], multiple-filled skutterudites with an enhanced *zT* over 1.4 have been developed [6,7]. Together with the filling approach, formation of multiple phases beyond the grain size reduction is commonly employed since this approach can reduce the *κ*_lat_ and/or improve the power factor (*S*^2^*σ*) [8]. The *zT* of TE materials with multiple phases is not determined by the average transport properties of the individual phases. Various interfacial effects such as carrier filtering and boundary phonon scattering to explain *zT* enhancement have been proposed [6,7,8,9]. Introducing nanoparticles into the skutterudite matrix is one of the simplest routes to form materials with multiple phases, whereby a reduced *κ*_lat_ is obtained while maintaining the power factor of the skutterudite matrix [10,11]. However, a well-controlled fabrication process for uniform dispersion of nanoparticles in the skutterudite matrix is always required to secure the enhancement of *zT*.

The second technology to generate materials with multiple phases includes the solid-state phase transformation methods such as precipitation, separation, and eutectic transformation. In PbTe-based TE alloys, this approach has been widely used with decomposition during the cooling process and resulted in the formation of nanocomposites with uniformly distributed nanoinclusions [12,13]. Recently, several studies for the preparation of bulk-type materials with multiple phases have been reported in La-, Ce-, and Yb-filled skutterudites mainly due to the uneven distribution of cations [14,15,16,17,18].

Here we provide a rapid solidification process (RSP)-based synthesis route to prepare submicron-scale heterograin structure of *p*-type Yb-filled (Fe,Co)_4_Sb_12_ skutterudites since RSP can trigger the generation of non-equilibrium or supersaturated phases due to high quenching speed. In this study, we used a melt spinning (MS) process, in which cooling rates are in the range of 10^4^–10^7^ K s^−1^.

Mixed grains of Yb_x_Fe_3−y_Co_1+y_Sb_12_ and Yb_z_Fe_3+y_Co_1−y_Sb_12_ were formed in melt spun ribbons, and this heterograin structure was maintained in the compacted bulks. A high power factor of ~3.13 mW m^−1^ K^−2^ and a low *κ*_lat_ of ~1.14 W m^−1^ K^−1^ were observed at 700 K in Yb_0.85_Fe_3_CoSb_12_ (nominal composition) in the presence of an interface between two different grains. A peak *zT* value of 0.66 at 700 K was obtained.

## 2. Materials and Methods

Ingots of Yb_x_Fe_3_CoSb_12_ (x = 0.80, 0.85, 0.90) were prepared by melting and solidification. Mixtures of high purity (>99.99%) elements of Yb, Fe, Co, and Sb with targeted compositions were melted at 1373 K for 12 h in a vacuum (at 2.0 × 10^−2^ Pa) sealed quartz tube (15 mm in diameter; inside wall was coated with carbon by acetone cracking). Acquired ingots were crushed into chunks (~3 mm), and then the ribbons (1–1.5 mm wide, 10–15 mm long, and 10–12 μm thick) were fabricated by using the melt spinning (MS) process. The MS equipment consists of an induction heater, a gas control system, a graphite nozzle (~0.5 mm), and a Cu wheel (250 mm in diameter). The crushed chunks (~7 g) of ingot were melted in a graphite nozzle by induction heating, and molten alloy was injected onto a rotating Cu wheel (~3600 rpm). The ribbons were pulverized into powders in an agate mortar, and compacted bulks (10 mm in diameter and 3 mm in thickness) were fabricated by using spark plasma sintering (SPS, SPS-630lx, Fuji Electronic Industrial, Saitama, Japan) at 823 K for 3 min under a uniaxial pressure of 45 MPa.

Phase formation behaviors of the melt spun ribbons and SPSed bulks were analyzed by the X-ray diffraction (XRD) method (EMPYRENA diffractometer, PANalytical B.V., The Netherlands) with Cu K_α1_ radiation. The microstructures of the samples were confirmed by field emission scanning electron microscopy (FESEM, JSM-7500F, JEOL, Tokyo, Japan). In order to determine the *zT* values of the SPSed bulks, we evaluated the temperature dependences of *σ* and *S* by commercial equipment (ZEM-3, ULVAC, Chigasaki, Japan). The temperature dependence of *κ* (=*C*_p_ × *ρ* × *λ*) was calculated from a separate measurement of the heat capacity (*C*_p_), density (*ρ*), and thermal diffusivity (*λ*). The *C*_p_ values were measured by using differential scanning calorimetry (DSC 200 F3 Maia, NETZSCH, Selb, Germany) and *λ* values were measured by using a laser flash apparatus (LFA467, NETZSCH, Selb, Germany) from 300 K to 700 K. In order to evaluate the electronic and thermal transport parameters, we also obtained the carrier concentration (*n*_c_) and Hall mobility (*μ*_Hall_) at 300 K by using a Hall measurement system (HMS-3000, Ecopia, Chandler Heights, AZ, USA) with a van der Pauw configuration.

## 3. Results and Discussion

**Figure 1:**
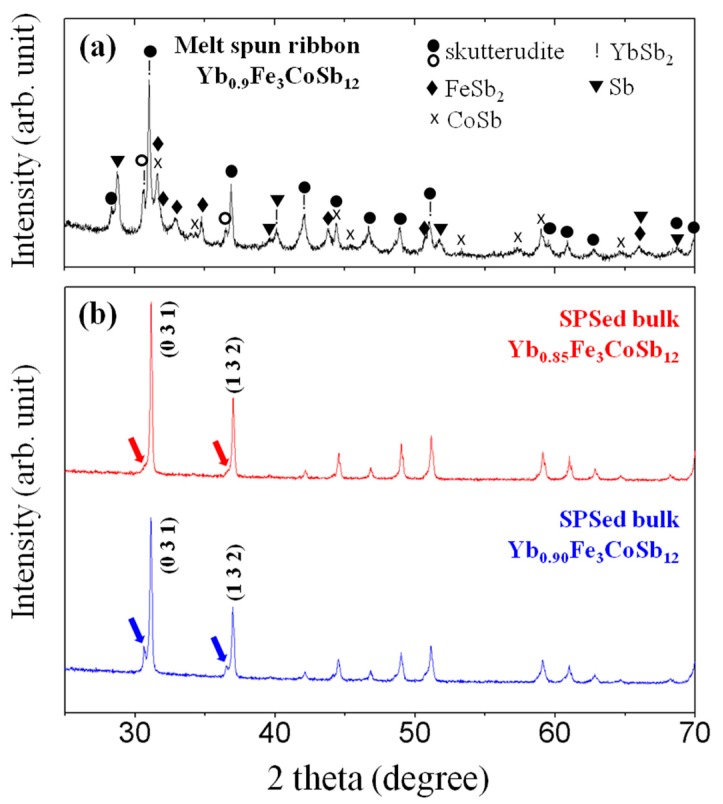
(**a**) X-ray diffraction (XRD) pattern for the melt spun ribbons of Yb_0.90_Fe_3_CoSb_12_ and (**b**) XRD patterns for the SPSed bulks of Yb_0.85_Fe_3_CoSb_12_ and Yb_0.90_Fe_3_CoSb_12_.

**Figure 2:**
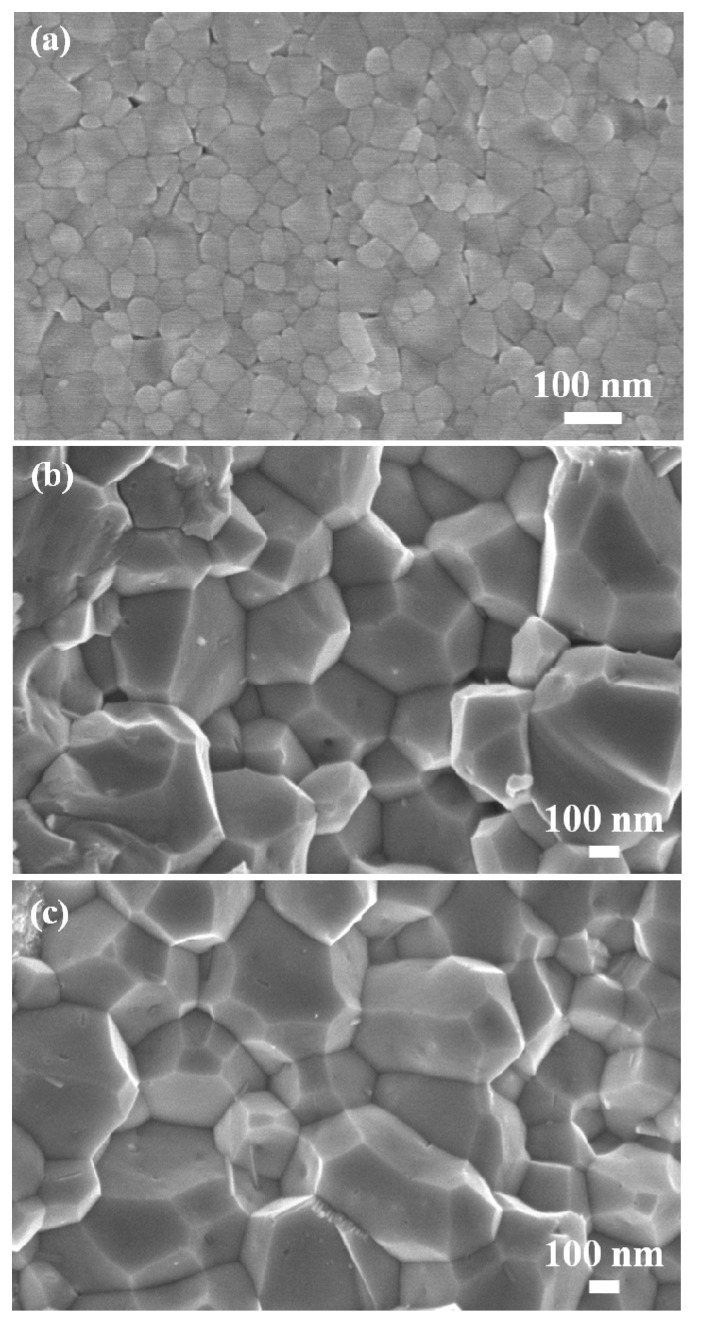
(**a**) Scanning electron microscopy (SEM) image for the contact surface of melt spun ribbon of Yb_0.85_Fe_3_CoSb_12_; and SEM images for the fractured surfaces of SPSed, (**b**) Yb_0.85_Fe_3_CoSb_12_ and (**c**) Yb_0.90_Fe_3_CoSb_12_ bulks.

By using the MS process, we attempted to generate *p*-type Yb-filled (Fe,Co)_4_Sb_12_ skutterudites with multiple phases. Figure 1a shows the XRD analysis results for the melt spun ribbons of Yb_0.9_Fe_3_CoSb_12_ (starting nominal composition). The melt spun ribbons of Yb_0.9_Fe_3_CoSb_12_ contain multiple secondary phases including FeSb_2_, CoSb, YbSb_2_, and Sb. It should be noted that two phases of CoSb_3_-based alloys (skutterudites) with different lattice constants were detected. Figure 1b shows the XRD patterns of SPSed Yb_x_Fe_3_CoSb_12_ (x = 0.85, 0.90) bulks. Yb-filled skutterudites without any impurities were successfully fabricated, benefitting from the activated phase evolution during SPS due to the homogenous dispersion of various nanoscale phases in the ribbons (the sizes of grains (Yb filled Fe_3_CoSb_12_, FeSb_2_, CoSb, YbSb_2_, and Sb ranged from 50 nm to 100 nm, as shown in SEM image (Figure 2a) of the contact surface of melt spun ribbon of Yb_0.85_Fe_3_CoSb_12_, which triggers the diffusion of ions during the sintering process) [19,20,21], while two different Yb-filled skutterudites found in melt spun ribbons still remained in SPSed bulks. Distinct left shoulders (red arrows in Figure 1b) can be seen in all XRD peaks of Yb_0.85_Fe_3_CoSb_12_, and separated smaller XRD peaks (blue arrows in Figure 1b) of the (0 3 1) and (1 3 2) reflections are clearly observed in Yb_0.9_Fe_3_CoSb_12_. These results suggest that a composite of two different skuttrudites was generated by MS and SPS due to uneven distribution of cations, and the formation of two different filled skutterudites has been reported previously [14,15,16,17,18]. The calculated lattice constants of the majority skutterudite (9.0820(1) Å for Yb_0.85_Fe_3_CoSb_12_ and 9.0881(7) Å for Yb_0.9_Fe_3_CoSb_12_) are smaller than those of the minority skutterudite (9.1659(5) Å for Yb_0.85_Fe_3_CoSb_12_ and 9.2037(9) Å for Yb_0.9_Fe_3_CoSb_12_), indicating that the compositions of the composite are Yb_x_Fe_3−y_Co_1+y_Sb_12_ (Co-rich skutterudite) and Yb_z_Fe_3+y_Co_1−y_Sb_12_ (Fe-rich skutterudite), related with the size difference between Fe^2+^ (CN = 6, 0.61 Å) and Co^3+^ (CN = 6, 0.55 Å) [22]. In addition, the lattice constants of Yb_x_Fe_3−y_Co_1+y_Sb_12_ are almost the same, while those of Yb_z_Fe_3+y_Co_1−y_Sb_12_ increase gradually with Yb content due to the charge stability in *p*-type skutterudites.

Figure 2b,c shows SEM images of the fractured surface of SPSed bulks of Yb_0.85_Fe_3_CoSb_12_ and Yb_0.9_Fe_3_CoSb_12_ with ~500 nm average grain size. It is noted that features of nano-scale inclusions are not observed, indicating that a submicron-scale heterograin structure is fabricated in compacted bulks.

**Figure 3:**
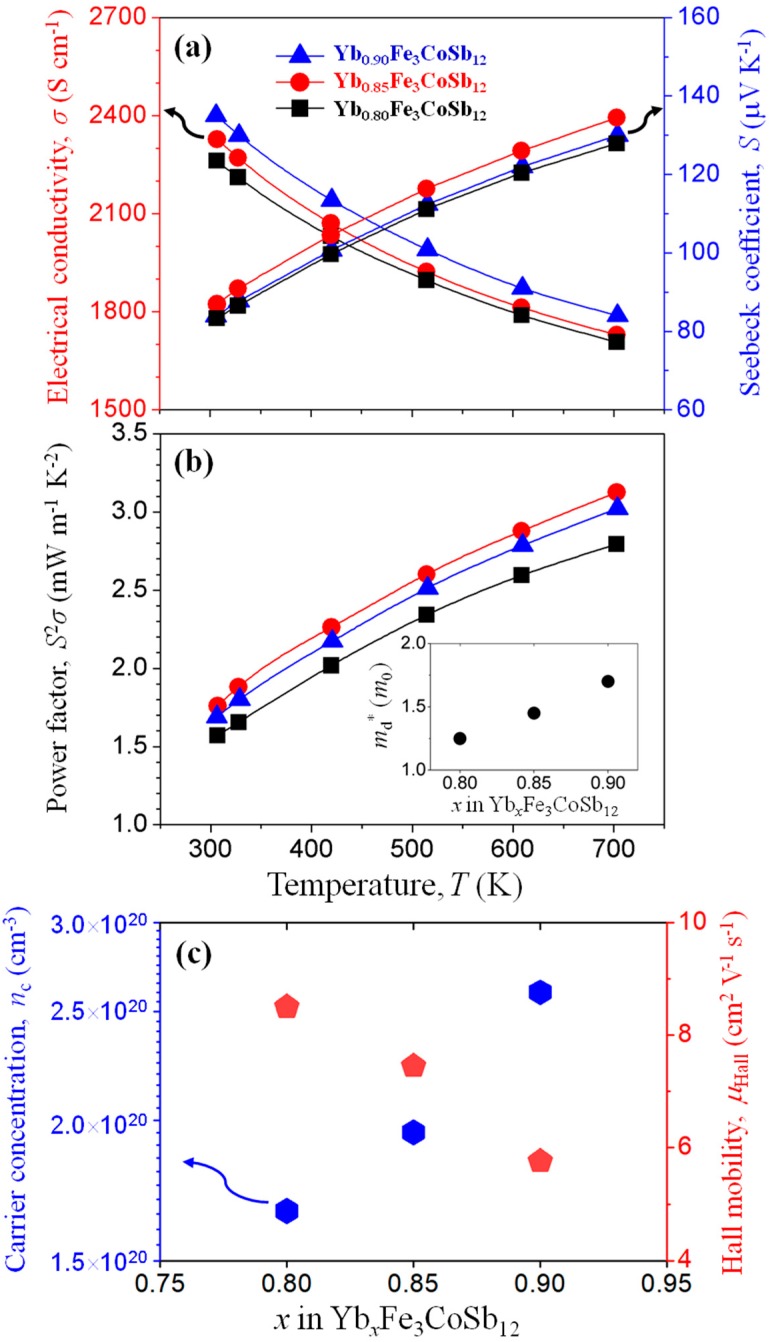
Temperature dependences of (**a**) electrical conductivity (*σ*), Seebeck coefficient (S) and (**b**) power factor (*σS*^2^) for Yb_x_Fe_3_CoSb_12_ (x = 0.80, 0.85, 0.90) bulks. Density of states effective mass (m_d_*) values are shown in the inset of (**b**). (**c**) Room temperature carrier concentration (*n*_c_) and Hall mobility (*μ*_Hall_) for Yb_x_Fe_3_CoSb_12_ (x = 0.80, 0.85, 0.90) bulks.

Figure 3a shows the temperature-dependent *σ* and *S* of SPSed Yb_x_Fe_3_CoSb_12_ (x = 0.80, 0.85, 0.90) bulks, respectively. All *S* values were found to be positive, confirming *p*-type semiconducting characteristics. Both the *σ* and *S* values slightly increase with Yb content within the entire measured temperature range, and enhanced power factor (*S*^2^*σ*) values were obtained at higher Yb content (x = 0.85 and 0.90), as shown in Figure 3b. To clarify this, we obtained the *n*_c_ and *μ*_Hall_ at 300 K form Hall measurement and also calculated the density of states (DOS) effective mass (m_d_*) by the following Equation (2) [2]:

(2) $$S=\frac{8\pi^{2}\;k_{B}{}^{2}}{3eh^{2}}{\left(\frac{\pi }{3n_{c}}\right)}^{2/3}m_{d}^{*}T,$$

where *k_B_* is the Boltzmann constant, and *h* is the Planck constant, respectively. As shown in Figure 3c, the *n*_c_ values increase with Yb content, which is further experimental evidence for the generation of a heterograin structure. Since the Fe atom has one electron less than the Co atom (Fe_Co_^/^), the increase in *n*_c_ is considered to be related with the formation of Fe-rich skutterudite. By the energy filtering effect originating from the difference in band gap between heterograins, m_d_* values increase with Yb content (inset of Figure 3b). However, *μ*_Hall_ values decrease with Yb content, mainly due to the carrier scattering at the interface between Yb_x_Fe_3−y_Co_1+y_Sb_12_ and Yb_z_Fe_3+y_Co_1−y_Sb_12_ grains (Figure 3c). Related with this trade-off relationship between m_d_* and *μ*_Hall_, a maximum power factor value of ~3.13 mW m^−1^ K^−2^ is obtained in Yb_0.85_Fe_3_CoSb_12_, benefitting from the formation of a heterograin structure with optimized band alignment.

**Figure 4:**
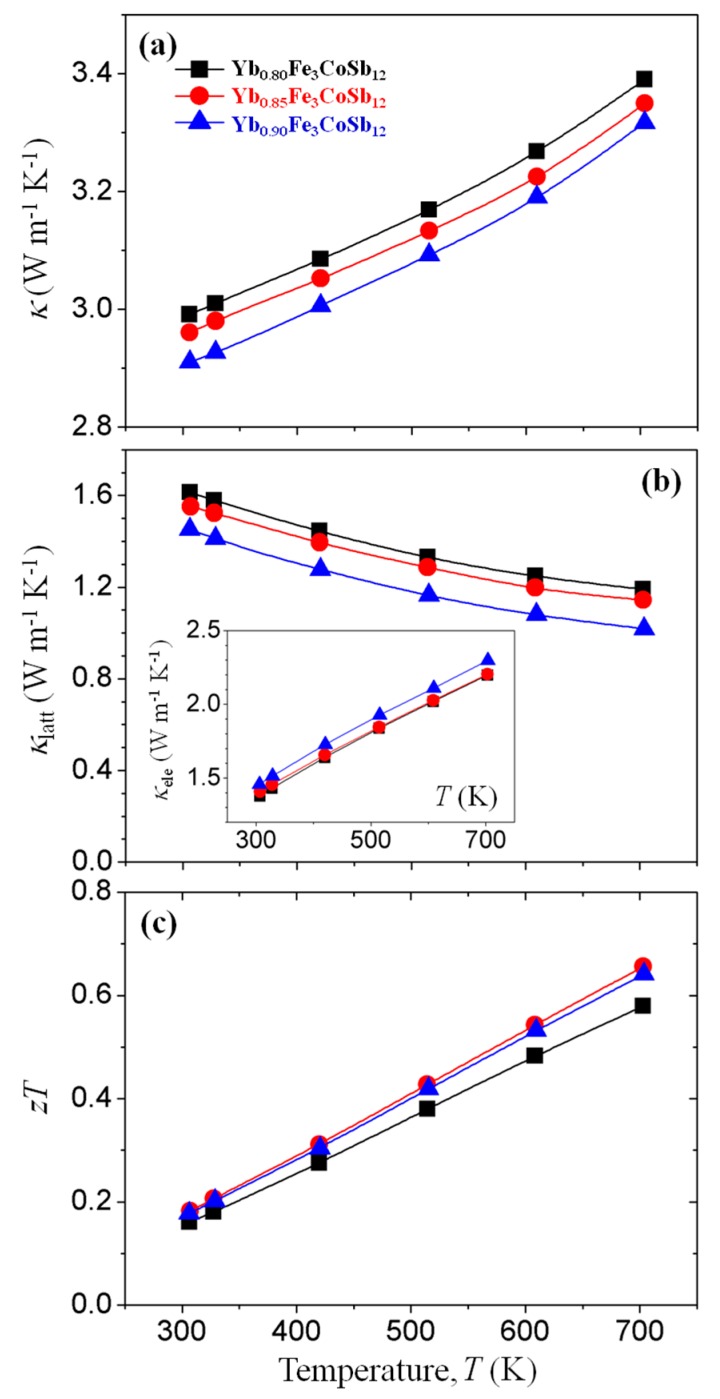
Temperature dependences of (**a**) total thermal conductivity (*κ*) and (**b**) lattice thermal conductivity (*κ*_lat_) for Yb_x_Fe_3_CoSb_12_ (x = 0.80, 0.85, 0.90) bulks. Electronic contribution for the thermal conduction (*κ*_ele_) is shown in the inset of (**b**). (**c**) Temperature dependences of dimensionless figure of merit (*zT*) for Yb_x_Fe_3_CoSb_12_ (x = 0.80, 0.85, 0.90) bulks.

We also elucidate the effect of the heterograin structure on the thermal transport properties of *p*-type Yb-filled skutterudites. As shown in Figure 4a, *κ* values of Yb_0.90_Fe_3_CoSb_12_ are rather lower compared to those of Yb_0.80_Fe_3_CoSb_12,_ despite higher *σ* values. We calculated the temperature-dependent *κ*_lat_ values with Yb content (Figure 4b) from measured *κ* and estimated *κ*_ele_ (inset of Figure 4b) by the Wiedemann-Frantz law (*κ*_ele_ = *LσT*, where *L* is Lorentz number).

Assuming that the acoustic phonon scattering mechanism is dominant, temperature-dependent *L* values are calculated by the following Equation (3) [23]:

(3) $$L=1.5+Exp\left[-\frac{\left|S\right|}{116}\right].$$

The *L* values ranged from 1.814 to 1.988 W Ω K^−2^ for all samples. The *κ*_lat_ values almost proportionally decrease with temperature, suggesting that the effect of the bipolar contribution (*κ*_bp_) on thermal conduction is negligible even at higher temperatures. A temperature-independent vibrational mode in *p*-type La-filled Fe_3_CoSb_12_ [24] is not found in this Yb-filled (Fe,Co)_4_Sb_12_ system. This difference in thermal conduction behavior might originate from the variable charge state of Yb ions. Resultantly, *κ*_lat_ values decrease with Yb content within the entire measured temperature range due to the intensified rattling effect and mismatched phonon modes at the interface between heterograins. Low *κ*_lat_ values of 1.55 W m^−1^ K^−1^ at 300 K and 1.14 W m^−1^ K^−1^ at 700 K were observed in Yb_0.85_Fe_3_CoSb_12_. Temperature dependence of z*T* is shown in Figure 4c. A peak *zT* of 0.66 was obtained at 700 K for Yb_0.85_Fe_3_CoSb_12_ due to the enhanced power factor and simultaneously reduced *κ*_lat_, thus demonstrating that heterograin structuring is a promising approach to improve the *zT* of filled skutterudites.

## 4. Conclusions

We fabricated *p*-type Yb-filled (Fe,Co)_4_Sb_12_ skutterudites with micro-scale heterograins by using a combined technique of melt spinning and spark plasma sintering. By the in-situ generation of interfaces between Fe-rich and Co-rich grains, which act as both an energy barrier for carrier transport and a scattering center for phonon transport, the power factor was enhanced due to the formation of an energy barrier for carrier transport, and the lattice thermal conductivity was reduced due to intensified boundary phonon scattering. This approach to prepare heterograin structured thermoelectric materials provides progress in both the facile process that is suggested and the fundamentals of defect engineered materials for enhancing thermoelectric performance.
